# Relation of Perceived Stigma to Adverse Events of Medications in Patients with Epilepsy

**DOI:** 10.1155/2016/5362806

**Published:** 2016-03-16

**Authors:** Ekaterina Viteva

**Affiliations:** Department of Neurology, University of Medicine, 15A Vassil Aprilov Street, 4002 Plovdiv, Bulgaria

## Abstract

*Purpose.* We aimed to assess the influence of adverse events (AEs) of antiepileptic drugs (AEDs) on perceived stigma of Bulgarian patients with epilepsy.* Methods.* Our study was based on questionnaires (Liverpool Adverse Events Profile (LAEP) and stigma scale), information from medical documentation, and an interview on clinical factors of 153 consecutive patients with epilepsy.* Results.* Perceived stigma was observed in 64.71% of the study participants. There was a significant association between perceived stigma and the total LAEP score (*p* < 0.05, *F* = 13.71). Patients who reported AEs had an increased risk of perceiving stigma compared to those who did not experience AEs. A significant correlation between perceived stigma and the presence of neurological and psychiatric AEs (*p* < 0.001, *r* = +0.60) and a mild correlation between perceived stigma and the presence of nonneurological AEs (*p* < 0.01, *r* = +0.20) were verified. In a multivariate regression analysis the only predictors of perceived stigma were AED polytherapy and the presence of neurological and psychiatric AEs.* Conclusions.* AEs of AEDs in patients with epilepsy significantly correlate with perceived stigma. Our study results will be useful in the campaign to overcome stigma predictors.

## 1. Introduction

Stigma was defined by Link and Phelan as a social process observed when there are elements of labeling, stereotyping, and discrimination due to previously specified characteristics, such as being different and unacceptable, which result in social status loss [1]. Health-related stigma is based on special characteristics of a health problem or state [2]. In persons with epilepsy, stigma is the result of unpredictability of seizures, social exclusion because of a negative attitude toward epilepsy patients from society, and difficulties in education, having a family, and finding a job [3]. People perceiving themselves as being stigmatized have feelings of devaluation, shame, secret, or withdrawal and fear of discrimination.

The use of antiepileptic drugs is frequently associated with adverse effects (AEs) such as idiosyncratic reactions, dose-related neurocognitive effects, and complications of long-term use. AEs are associated with treatment duration and effectiveness. Rho et al. [4], Elsharkawy et al. [5], and Perucca et al. [6] support that drug adverse events (particularly cognitive and neurological impairment) correlate more significantly with quality of life variations than other clinical characteristics (e.g., seizure frequency). Investigators have paid little attention to the influence of adverse events of medications on stigma in patients with epilepsy [7]. However, no study evaluating adverse events and stigma in patients with epilepsy has been performed in Bulgaria. The purpose of this paper is assessment of the correlation of adverse events of antiepileptic drugs with a perceived stigma in Bulgarian patients with epilepsy.

## 2. Patients and Methods

The study was performed with a representative sample of patients with epilepsy who attended the Clinic of Neurology at the University Hospital in Plovdiv, Bulgaria, for regular examinations, unsatisfactory seizure control, or adverse events from treatments.

All study procedures were performed after the approval of the Local Ethics Commission at the University of Medicine, Plovdiv. The study design was explained to every patient. Each patient signed an informed consent form before participating in the study procedures.

The study included 153 consecutive patients with epilepsy. We used the following inclusion criteria: a signed informed consent form; age between 18 and 65 years; a diagnosis of epilepsy; lack of cognitive impairment based on the rapid evaluation of cognitive functions [8] with a score <47 in patients up to 60 years of age and primary education or <46 in patients between 60 and 65 years of age and less than a primary education or illiteracy; lack of progressive somatic or neurological disease; stable treatment with antiepileptic drugs (AED) for at least 3 months prior to study entry (unchanged dose and type of drugs); lack of a simple or complex partial seizure in the last 4 hours; and lack of generalized tonic-clonic seizures in the last 24 hours. Patients with another progressive neurological or psychiatric disease or another chronic severe physical comorbidity (diabetes, asthma, heart, renal, or hepatic failure, etc.) on stable concomitant medication were not included in the study.

Data on demographics (age and gender), clinical findings (seizure type, type of epilepsy, etiology of epilepsy, seizure frequency, prescribed treatment, and adverse events) were collected by a trained health professional with an interview, examination of each patient's medical documentation, and the Liverpool Adverse Events Profile (LAEP) questionnaire. We determined the correlations between perceived stigma and the total score of LAEP, between perceived stigma and the presence of neurological and nonneurological AEs, and between the number of medications being taken and the LAEP total score.

Patients completed the Liverpool Adverse Events Profile [9] and stigma scale [10].

The frequency of AEs in the preceding 4 weeks was identified by the Liverpool Adverse Events Profile questionnaire, which is used as a systematic measure of the most common AEs of antiepileptic drugs. The variables recorded in LAEP include 19 self-reported symptoms. They were rated by each patient on a 4-point scale: 1 indicates that the symptom is never a problem; 2 indicates that it is rarely a problem; 3 indicates that it is sometimes a problem; and 4 indicates that it is always or often a problem (theoretical). Thus, it is possible to analyze the scores of individual symptoms as well as calculate an overall symptom score. The instrument is available in a 19-item version and a 21-item version [9]. The ratings of each item are added up to yield a total score, which is indicative of the total burden of AEs. The reliability and validity of the LAEP, which has been widely used in epilepsy research [11–15], have been demonstrated in a large European study [9]. It is assumed that, in cases with a total score of at least 45, there is a significant probability of drug toxicity. The questionnaire was validated in Bulgarian by Kuzmanova et al., who created two subscales “Neurological and Psychiatric Side Effects” (unsteadiness, tiredness, headache, double/blurred vision, difficulty concentrating, shaky hands, dizziness, sleepiness, memory problems, disturbed sleep, restlessness, and feelings of aggression, nervousness/agitation, and depression) (theoretical score range 24–56) and “Nonneurological Side Effects” (hair loss, skin problems, upset stomach, trouble with mouth/gums, and weight gain) (theoretical score range 5–20). The Bulgarian version of LAEEP showed high internal consistency and reliability (Cronbach's *α* 0.86, test-retest reliability higher than 0.75). The creation of two subscales “Neurological and Psychiatric Side Effects” and “Nonneurological Side Effects” showed good internal consistency (Cronbach's *α* of 0.85 and 0.71, resp.). The Bulgarian version of LAEP scores significantly correlated with other questionnaires such as the Quality of Life in Epilepsy Inventory-89 (QOLIE-89) and showed a good discriminative validity between groups with different levels of self-assessed AEs of AED [16].

The stigma scale consists of three questions pertaining to a patient's opinions about the attitudes of people. The possible answers are “yes” and “no.” Severity of stigmatization depends on the number of positive answers. The scale scores are 0 = “none,” 1 positive answer = “mild perceived stigma,” 2 positive answers = “moderate,” and 3 positive answers = “severe” (final score 0–3). If there was one affirmative response, we accepted the patient as stigmatized. The scale has been used by Atadzhanov et al. in patients with epilepsy and is known to have satisfactory convergent validity and internal consistency [2].

The collected primary information was checked, encoded, and entered into a computer database for statistical analysis. Data were processed using STATA Version 10 (Stata Corp., College Station, TX, USA) and SPSS (Statistical Package for the Social Sciences) version 14.0 (SPSS Inc., Chicago, IL, USA). The results for quantitative variables are expressed as the mean ± SE (standard error) and results for qualitative variables are expressed as percentages ± SE. Data were processed using SPSS 17.0. Cross tabulation analysis was used to determine correlations between perceived stigma and AE from antiepileptic treatment. The complex influence of the significant findings was determined using a multivariate logistic regression analysis (regression coefficient (*B*)). Significance was set at *p* < 0.05.

## 3. Results

Fifty-nine (38.6%) participants in our study were men. The remaining 94 participants (61.4%) were women. Their mean age was 39.34 ± 1.01 years. The clinical findings of the participants are shown in Table 1.

According to the results from the stigma scale, 99 (64.7%) of the patients had a perceived stigma. Seven (4.6%) of the participants had a mild perceived stigma, 28 (18.3%) had a moderate perceived stigma, and 64 (41.8%) had a severe perceived stigma.

The mean LAEP score was 29.19 ± 0.75. The mean number of reported adverse events was 8.43 ± 0.34. Nonneurological AEs were reported by 32 (20.9%) participants, neurological and psychiatric AEs were reported by 90 (58.8%), and neurological, psychiatric, and nonneurological AEs were reported by 30 (19.6%). According to the LAEP results, there was a significant probability of toxicity in 12 (7.8%). In these cases antiepileptic treatment was modified.

We found a significant association between perceived stigma and the total LAEP score, *p* < 0.05 (*F* = 13.71). The patients with moderate and severe perceived stigma had much more frequent AEs than those without perceived stigma or with mild perceived stigma (Table 2).

We demonstrated a significant correlation between perceived stigma and the presence of neurological and psychiatric AE (*p* < 0.001, *χ* ^2^ = 57.76; *r* = +0.60, *p* < 0.001) (Table 3).

We discovered a mild correlation between perceived stigma and the presence of nonneurological AE (*p* < 0.05, *χ* ^2^ = 7.79; *r* = +0.20, *p* < 0.01) (Table 4).

The multivariate regression analysis demonstrated that the only predictors of perceived stigma were AED polytherapy (*p* < 0.001; OR = 20.864; 95% CI 8.882–49.011) and the presence of neurological and psychiatric AE (OR = 11.038, 95% CI 5.046–24.162). They explained 51% of perceived stigma variations *p* < 0.001 (*F* = 84.86) (Table 5).

## 4. Discussion

The negative impact of epilepsy on all social aspects and the phenomenon of stigmatization have been thoroughly investigated and discussed by many scientists. Efforts to minimize the unfavorable effects of stigma explain the persisting interest in its determinants.

The purpose of our study was to assess the relation between adverse events of antiepileptic drugs and perceived stigma in Bulgarian patients with epilepsy. The role of medication AEs has been somehow underestimated by previous investigations. A study by Taylor et al. is the only study that described a correlation of the LAEP score with felt stigma (*p* < 0.001; *r* = 0.45) [7]. The authors concluded that the participants who felt being stigmatized reported more adverse events from their treatment. Based on the multivariate linear regression analysis, LAEP scores proved to be one of the significant predictors of felt stigma *p* = 0.001 (*β* = 0.13) [7]. Our study results confirmed the significance of LAEP scores for perceived stigma. Moreover, we discovered an association between AED adverse events and the severity of perceived stigma. The patients with moderate and severe perceived stigma reported AE much more frequently than those without perceived stigma or with mild perceived stigma. Another important issue is that our study results emphasized the significant association of some specific AE (neurological and psychiatric) with perceived stigma as well as their predictive role.

## 5. Limitations

The first limitation of our study is that we investigated perceived stigma of patients with epilepsy. Further investigations are needed to examine the relation of AE not only to perceived stigma but also to enacted stigma, as well as actual attitudes and reported behavior of the Bulgarian community toward patients with epilepsy. The participation of only those with access to the University Clinic of Neurology, who attended it for regular examinations, unsatisfactory seizure control, or adverse events from treatment, is also a limitation. Further investigations of patients with diverse demographics and clinical and social characteristics are needed.

In* conclusion*, the modifiability of drug adverse events and the significant correlation with perceived stigma (especially of neurological and psychiatric adverse events) will provide another reason to focus interventions on efficiently reducing AEs to overcome stigma predictors in patients with epilepsy.

## Figures and Tables

**Table 1:** Clinical findings of study participants.

Clinical findings	*N*	*p* (%)	SE
Type of epilepsy			
Partial	99	64.7	3.88
Generalized	54	35.3	3.88
Type of seizures			
(1) Partial			
Simple partial	2	1.3	—
Complex partial	10	6.5	2.01
Partial with secondary generalization	34	22.2	3.37
(2) Generalized			
Generalized tonic-clonic	52	34.0	3.84
(3) Polymorphic	55	36.0	3.89
Etiology of epilepsy			
Idiopathic	46	30.0	3.72
Cryptogenic	55	36.0	3.89
Symptomatic	52	34.0	3.84
Recent seizure frequency			
No seizures in recent years	46	30.1	3.72
Low (1 seizure/year or 1–11 seizures/year)	21	13.7	2.79
High (per month, per week, or daily)	86	56.2	4.02
Treatment			
Monotherapy	54	35.3	3.88
polytherapy	99	64.7	3.88
Adverse events (AEs)			
No	61	39.9	3.97
Yes	92	60.1	3.97

**Table 2:** Association of perceived stigma with the total score of LAEP.

Stigma	*N*	Mean	SE	CI		Min	Max
				Lower bound	Upper bound		
No	54	23.52	0.945	19.62	25.41	0	47
Mild	7	21.86	0.857	19.76	23.95	21	27
Moderate	28	31.04	1.887	27.16	34.91	21	56
Severe	64	33.05	1.229	30.59	35.50	0	55
Total	153	28.80	0.790	27.24	30.36	0	56

**Table 3:** Correlation between perceived stigma and the presence of neurological and psychiatric AEs.

Stigma	Neurological and psychiatric AEs		
	No	Yes	Total
No *N* (*p*%)	41 (75.9%)	13 (24.1%)	54 (100%)
Mild *N* (*p*%)	6 (75.9%)	1 (14.3%)	7 (100%)
Moderate *N* (*p*%)	9 (32.1%)	19 (67.9%)	28 (100%)
Severe *N* (*p*%)	7 (10.9%)	57 (89.1%)	64 (100%)
Total *N* (*p*%)	63 (41.2%)	90 (58.8%)	153 (100%)

**Table 4:** Correlation between perceived stigma and the presence of nonneurological AEs.

Stigma	Nonneurological AEs		
	No	Yes	Total
No *N* (*p*%)	48 (88.9%)	6 (11.1%)	54 (100%)
Mild *N* (*p*%)	7 (100%)	0 (0%)	7 (100%)
Moderate *N* (*p*%)	20 (71.4%)	8 (28.6%)	28 (100%)
Severe *N* (*p*%)	46 (71.9%)	18 (28.1%)	64 (100%)
Total *N* (*p*%)	121 (79.1%)	32 (20.9%)	153 (100%)

**Table 5:** Results from the multivariate regression analysis on perceived stigma predictors.

Model	*B*	SE	Beta	*t*	*p*
Constant	0.134	0.128		1.045	0.298
Polytherapy	0.494	0.068	0.494	7.246	0.000
Neurological and psychiatric AEs	0.270	0.131	0.278	2.056	0.042
Nonneurological AEs	−0.056	0.090	−0.048	−0.623	0.534
LAEP	0.028	0.115	0.036	0.242	0.809

## Appendix

*Stigma Scale*

1. Do you feel other people are uncomfortable with you because of your disease?
2. Do you feel other people treat you like an inferior person?
3. Do you feel other people would prefer to avoid you?

*Liverpool Adverse Events Profile.* During the last four weeks have you had any of the problems listed below?

- unsteadiness
  □ (4) Always or often a problem
  □ (3) Sometimes a problem
  □ (2) Rarely a problem
  □ (1) Never a problem
- tiredness
  □ (4) Always or often a problem
  □ (3) Sometimes a problem
  □ (2) Rarely a problem
  □ (1) Never a problem
- restlessness
  □ (4) Always or often a problem
  □ (3) Sometimes a problem
  □ (2) Rarely a problem
  □ (1) Never a problem
- feelings of anger or aggression to others
  □ (4) Always or often a problem
  □ (3) Sometimes a problem
  □ (2) Rarely a problem
  □ (1) Never a problem
- nervousness and/or agitation
  □ (4) Always or often a problem
  □ (3) Sometimes a problem
  □ (2) Rarely a problem
  □ (1) Never a problem
- headache
  □ (4) Always or often a problem
  □ (3) Sometimes a problem
  □ (2) Rarely a problem
  □ (1) Never a problem
- hair loss
  □ (4) Always or often a problem
  □ (3) Sometimes a problem
  □ (2) Rarely a problem
  □ (1) Never a problem
- problems with skin (e.g., acne, rash)
  □ (4) Always or often a problem
  □ (3) Sometimes a problem
  □ (2) Rarely a problem
  □ (1) Never a problem
- double or blurred vision
  □ (4) Always or often a problem
  □ (3) Sometimes a problem
  □ (2) Rarely a problem
  □ (1) Never a problem
- upset stomach
  □ (4) Always or often a problem
  □ (3) Sometimes a problem
  □ (2) Rarely a problem
  □ (1) Never a problem
- difficulty in concentrating
  □ (4) Always or often a problem
  □ (3) Sometimes a problem
  □ (2) Rarely a problem
  □ (1) Never a problem
- trouble with mouth or gums
  □ (4) Always or often a problem
  □ (3) Sometimes a problem
  □ (2) Rarely a problem
  □ (1) Never a problem
- shaky hands
  □ (4) Always or often a problem
  □ (3) Sometimes a problem
  □ (2) Rarely a problem
  □ (1) Never a problem
- weight gain
  □ (4) Always or often a problem
  □ (3) Sometimes a problem
  □ (2) Rarely a problem
  □ (1) Never a problem
- dizziness
  □ (4) Always or often a problem
  □ (3) Sometimes a problem
  □ (2) Rarely a problem
  □ (1) Never a problem
- sleepiness
  □ (4) Always or often a problem
  □ (3) Sometimes a problem
  □ (2) Rarely a problem
  □ (1) Never a problem
- depression
  □ (4) Always or often a problem
  □ (3) Sometimes a problem
  □ (2) Rarely a problem
  □ (1) Never a problem
- memory problems
  □ (4) Always or often a problem
  □ (3) Sometimes a problem
  □ (2) Rarely a problem
  □ (1) Never a problem
- disturbed sleep
  □ (4) Always or often a problem
  □ (3) Sometimes a problem
  □ (2) Rarely a problem
  □ (1) Never a problem
